# Rolling Into Trouble: A Case of Ben Wa Ball Retention in an Elderly Female Patient

**DOI:** 10.7759/cureus.75955

**Published:** 2024-12-18

**Authors:** Tanner C Carlock, David N Dhanraj

**Affiliations:** 1 Obstetrics and Gynecology, Wright State University Boonshoft School of Medicine, Dayton, USA

**Keywords:** age and sex, ben wa balls, dangerous sexual practice, kegel exercise, sex ed, sex-interest, sexual experimentation

## Abstract

Ben Wa balls are often used for sexual pleasure and pelvic floor exercise. However, their use can lead to complications, including retention within the vagina. We present a case of a 64-year-old female, status post-hysterectomy 20 years prior, who experienced the loss of a Ben Wa ball during sexual activity. Despite multiple retrieval attempts, the ball remained retained until she was transferred to a facility with an obstetrician-gynecologist (OB-GYN), who was able to successfully retrieve the lost ball.

## Introduction

Historically, people have used Ben Wa balls, love balls, orgasm balls, or vaginal balls for sexual pleasure and to strengthen their pelvic floor muscles. While their history is not well documented, some report their origins in ancient Japanese culture, approximately 500 AD [1]. Perhaps related to recent mentions on television programs such as Sex and the City, Broad City, and Fifty Shades Darker, the popularity of these balls has grown [2].

The pelvic floor musculature that Ben Wa balls attempt to strengthen has multiple functions, including supporting the pelvic organs, helping with control of bladder and bowel function, and contributing to sexual function. When inserted into the vagina, Ben Wa balls provide resistance as the pelvic floor muscles contract around them, which can strengthen the muscles over time. Strengthening these muscles can enhance sexual function through improved muscle control and tone.

Scientific evidence for enhancing sexual pleasure and strengthening pelvic floor musculature is lacking, and the opinions of Ben Wa balls are varied [3]. Some report significant pleasure, and others report no more than the sensation of a tampon [4]. The intended benefit of sexual pleasure and enhancement is achieved by placing the balls into the vagina and experimenting with a variety of movements. Anciently, women would rock on a chair and allow the balls to move back and forth, creating pleasure [1].

Ben Wa balls come in a variety of sizes and materials. Some are made of metal, while others are plastic with metal ball bearings inside. They also often come attached with a silicone or nylon cord mechanism for easy removal [4]. While the cord mechanism design allows for easy insertion and removal, complications may occur, including vaginal lacerations, perforation, edema, bleeding, and infection. This case highlights a rare but significant complication associated with the use of Ben Wa balls.

## Case presentation

**Figure 1:**
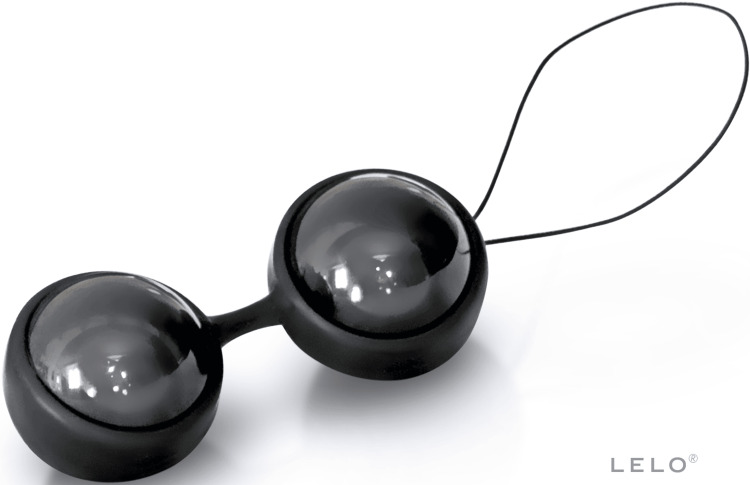
An image of the Ben Wa ball product used by the patient. LELO Beads Noir product website. Image: Permission to use this image was obtained from LELO, the original publisher [5].

A 64-year-old female presented to the emergency department (ED) after losing a Ben Wa ball in her vagina during sexual activity with her spouse. See Figure 1 for an image of the Ben Wa ball product used by our patient [5]. The patient had undergone a total hysterectomy approximately 20 years prior, from which she had no long-standing complications. She was regularly sexually active and reported a medical history of hypertension, gastroesophageal reflux disease, and obesity (BMI 41). 

The Ben Wa balls had been inserted in the vagina approximately three hours prior. During sexual activity, the silicone holding device broke and one ball was retained within the vaginal canal. She reported that the ball did not break and was intact. She was not in any significant discomfort at rest but did feel some mild "pressure." She reported that she had acquired the device online and that it was a plastic ball with a metal ball bearing inside.

The patient and her partner attempted to retrieve the ball at home but were unsuccessful. Those attempts caused her mild discomfort. In the ED, an emergency physician also failed to retrieve the object. The patient reported that the exam was very uncomfortable. No additional studies or imaging were performed. Due to a lack of on-call obstetrician-gynecologist (OB-GYN) specialists at that ED, the patient was transferred to a facility with on-call OB-GYN capabilities.

Following the transfer, she was examined by an OB-GYN in the ED room without any anesthesia. A pelvic examination was done with a Graves speculum and a small amount of lubricant. A uniformly smooth spherical object was identified in the posterior vagina at the edge of the vaginal cuff. The vaginal length from the introitus to the front of the object was approximately 6 cm. Thankfully, prior attempts at retrieval had not caused any significant injury or edema. However, the patient did have vaginal atrophy and moderate discomfort, which complicated the exam. The object was large enough (2.9 cm), the introitus narrow enough (approximately 5 cm), and the patient’s discomfort significant enough to limit the ability to open a speculum wide enough to meet the diameter of the ball. The speculum was removed, and a gloved finger was inserted without any additional lubricant. Ring forceps were then inserted slowly along the gloved finger. Attempts to grasp the object with the ring forceps were unsuccessful due to its smooth nature. 

A gloved finger was then utilized to maneuver the object slightly anteriorly. With this repositioning, ring forceps were reintroduced and successfully maneuvered around the ball. With careful manipulation, the forceps grasped the ball, facilitating its removal from the vaginal canal. Notably, the vaginal walls and vaginal cuff showed no signs of trauma, edema, or injury post-removal.

The patient was monitored and discharged from the ED with counseling on safe practices regarding the use of sexual enrichment aids. Despite counseling that Ben Wa balls are safe in the appropriate situation, she was adamant that she would not use them again. At a four-week follow-up appointment, the patient reported no complications, felt at her baseline level of health, and reported no additional issues with intimacy.

## Discussion

The use of items for sexual stimulation is extremely common, with one study indicating that nearly 80% of surveyed sexually active women 18-35 years old reported use [6]. Unfortunately, medical complications resulting from the use of these items are not uncommon. Multiple reports exist of devices used for sexual pleasure penetrating the vaginal wall or rectum [7,8]. In rare cases, these items may penetrate into the abdominal cavity, leading to potential intra-abdominal injury [9,10]. These complications can be life-threatening.

This case underscores the risks associated with the use of Ben Wa balls during intimacy. In our case, the device holding the ball in place broke, causing one ball to be lost in the vagina. Once identified, the smooth, rounded design of the ball complicated retrieval. Fortunately, the ball was made of a material that withstood manipulation using various instruments during removal. A less resistant material might have become damaged or broken, potentially injuring the patient and requiring surgical intervention. This is an important consideration, as Ben Wa balls have been known to be made of metal, plastic, silicone, or glass [11].

Healthcare providers need to be familiar with these items, know how to locate them, and have practical guidance on removal when they become dislodged. They should be prepared to employ various techniques for removal and understand their limitations. Thankfully, the object was identified on pelvic exam. X-ray, computed tomography, or ultrasound imaging may be necessary in more complex cases.

While intervention bedside was successful in our case, the patient did require transfer to another facility for removal. This resulted in prolonged time with the object inside, multiple exams, increased hospital exposure, and greater utilization of healthcare resources. If unable to be retrieved bedside, patients may require transfer to the operating room for removal under anesthesia, which incurs additional risk. While data are limited on retained vaginal foreign bodies, one study in the United Kingdom reported nearly 400 rectal foreign body removals are performed each year, many in the operating room, at an annual cost of £338,819 (~$450,000 USD) [12].

Additionally, healthcare providers should be familiar with the recommendations for when to seek medical attention. In this case, the patient sought attention within a few hours because of her fear related to the "lost" ball. In the absence of significant discomfort or bleeding, it would be reasonable not to suspect vaginal or intra-abdominal injury, and next-day follow-up in the clinic could have been a consideration. This scenario may be easily triaged if patients speak with their providers after hours. However, the patient's decision for when to seek care in the ED is challenging, especially in these anxiety-provoking moments. 

Healthcare providers must also be able to educate patients on the safe use of sexual enrichment aids. Unfortunately, many providers are not well-versed in offering this type of counseling. Evidence-based guidelines for counseling are needed to ensure that patients can safely engage in the use of sexual enrichment aids [13].

Finally, device manufacturers must ensure their products are made from high-quality materials. Care should be taken to select materials that will not cause tissue trauma or irritation while ensuring the product is resistant to breaking or shattering. Careful product design and manufacturing can go a long way to avoiding device complications.

## Conclusions

The retention of a Ben Wa ball in the vagina is a rare but significant complication. This case illustrates the challenges involved in the retrieval of a retained Ben Wa ball in an elderly female patient. It emphasizes the need for provider familiarity with sexual enrichment aids, patient education regarding their use, understanding of how to locate and retrieve them if complications arise, the ability to counsel patients on when to seek care, appropriate follow-up recommendations, and responsible product design by manufacturers.
